# The correlation between local weather and leptospirosis incidence in Kandy district, Sri Lanka from 2006 to 2015

**DOI:** 10.1080/16549716.2018.1553283

**Published:** 2019-01-09

**Authors:** N. D. B. Ehelepola, Kusalika Ariyaratne, Wasantha P. Dissanayake

**Affiliations:** a Department of Medicine, Teaching (General) Hospital – Kandy, Kandy, Sri Lanka; b Engineering Manager, Lanka Hydraulic Institute, Moratuwa, Sri Lanka

**Keywords:** Leptospirosis, rain, temperature, humidity, wavelet analysis

## Abstract

**Background**: Leptospirosis is an important public health problem in Sri Lanka. Most people become infected by contact with leptospires in soil and in surface water. Survival of leptospires in the environment depends upon the moisture in soil, humidity, temperature and surface water. Leptospires are spread by flood water and waterways. Therefore, the weather of an area influences the leptospirosis incidence of that area.

**Objectives**: To find out the correlations between the leptospirosis incidence in the district of Kandy, Sri Lanka, and local weather variables and then to explore the utility of the findings.

**Methods**: We gathered data on reported leptospirosis cases in the Kandy district and mid-year population data and calculated weekly incidences for 2006 to 2015. Daily weather data from Katugastota weather station was obtained and converted into weekly data. We plotted time series graphs and observed the correlation between six aggregated weather parameters and leptospirosis incidence. Those weather parameters were rainfall, the count of wet days per week, days with rainfall >100 mm per week, minimum temperature, average temperature and average humidity. Then we looked for correlations between leptospirosis incidence and those weather parameters by performing the wavelet analysis.

**Results**: Our wavelet analysis results show peaks of wet days per week, days with rainfall >100 mm per week, minimum temperature, average temperature and average humidity respectively after 2, 3, 13, 20 and 1 week lags were followed by peaks of leptospirosis incidence. Nadirs (troughs) of rainfall after a week were followed by nadirs of leptospirosis incidence.

**Conclusions**: All weather parameters studied are correlated with local leptospirosis incidence and the climate in Kandy is conducive for leptospirosis transmission. Leptospirosis incidence in the Kandy district is high compared to the national and global incidence. Therefore, leptospirosis preventive work in Kandy deserves more attention, especially during months with favorable weather for leptospirosis transmission.

## Background

Leptospirosis is a neglected emerging bacterial infection and already probably the most prevalent as well as most widely distributed zoonosis worldwide [1–6]. Leptospirosis has a wide spectrum of clinical manifestations and its severe form with multi organ failure (Weil’s disease) has a case fatality rate of > 40% [6]. It is an important public health problem and health problem of domestic animals, especially in tropical countries such as Sri Lanka [1–7]. Leptospirosis is endemic in Sri Lanka and has been a notifiable infection since 1991 [8]. Locally the disease is known as *Mee una* (rat fever).

Pathogenic leptospires (leptospira bacteria) mainly colonize the proximal tubules of nephrons of their natural host animals (mainly in rodents and domestic mammals) and are excreted in urine [2–6]. Pathogenic leptospires do not multiply outside the host animals (in the environment) [9]. Leptospires in the environment enter the body via abrasions or wounds in the epidermis or mucus membranes and even via intact mucus membranes and infect people (incidental hosts) [1–6]. People get infected when coming into contact with leptospires in moist soil, surface water or in rare cases direct contact with infected animals or their urine [1–6]. Desiccation (dehydration) kills leptospires in the environment [2,9]. If leptospires can survive for longer in the environment it enhances their chance of making contact with people [10]. Soil moisture, surface water, temperature and humidity influence the survival of leptospires in the environment and thus influence leptospirosis transmission [2–4,9,10]. Leptospires are spread via flood water and water ways [1,3,5,11]. Several outbreaks of leptospirosis following floods subsequent to heavy rainfall were reported from several countries and flooding was identified as a key driver of leptospirosis transmission in islands in Asia [3,5,11,12]. All of the above indicate that local weather affects the incidence of leptospirosis of a population.

There is a severe paucity of long-duration studies on correlations between leptospirosis incidence and multiple weather parameters globally and particularly from South Asia.

Considering the above background, we decided to study the correlation between weekly incidence of leptospirosis and local weather parameters of the Kandy district situated in the central highlands of Sri Lanka over a duration of 10 years (2006–2015).We expected the correlation between leptospirosis incidence and some weather parameters to be nonlinear and non-stationary based upon available information. For example, the rise of ambient temperature up to 34°C is favorable, but ambient temperature > 34°C is unfavorable for the survival of leptospires in the environment [13]. Whilst hot and rainy conditions are good for the survival of leptospires, during dry conditions with the same high temperature, soil is dried out faster and leptospires get killed. The wavelet time series analysis is reported as one of the most efficient method of studying non-stationary data [14–16]. To the best of our knowledge this is the first time this method of analysis was employed to determine the correlation between weekly leptospirosis incidence and multiple meteorological parameters in an Asian country.

### Study setting

**Figure 1.**
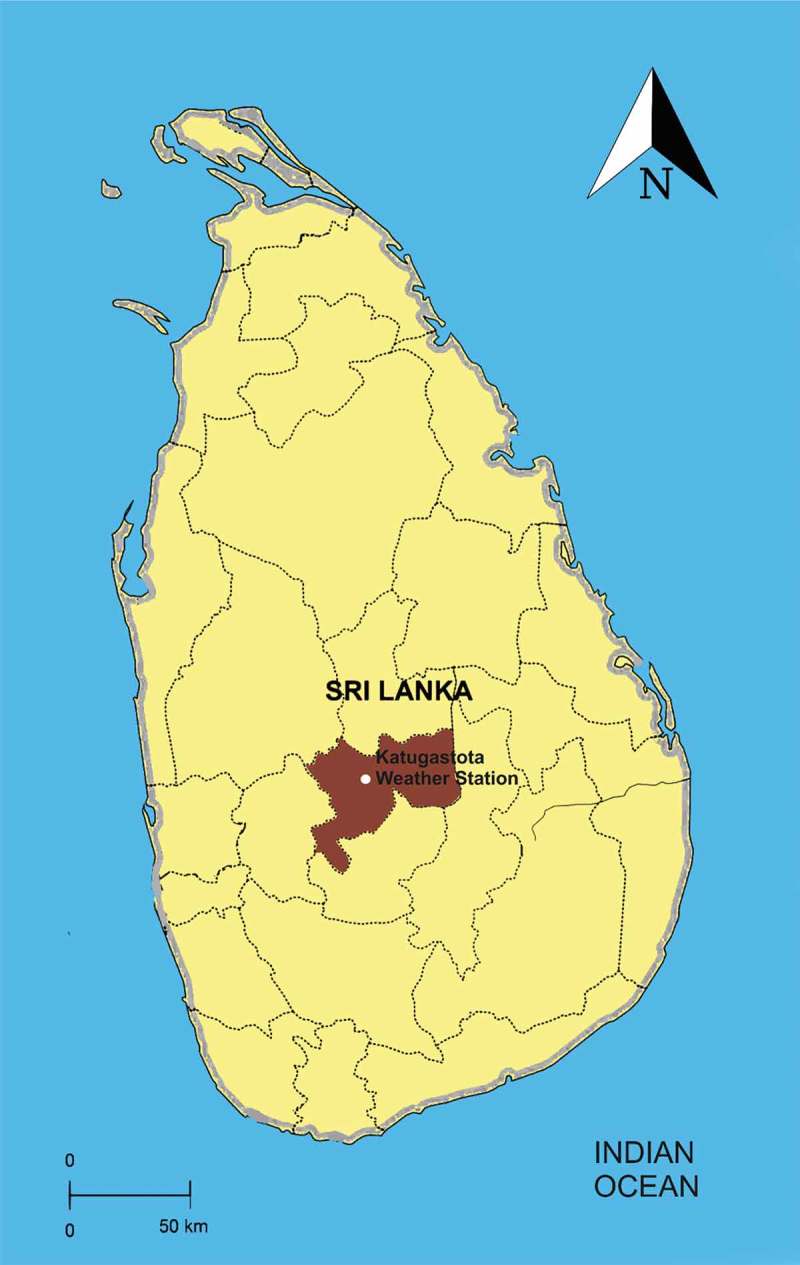
Our study area, Kandy district, on a map of Sri Lanka. Kandy district is shaded in light brown color in contrast to the other 24 districts. Location of the Katugastota weather stations from which we obtained our weather data is also depicted.

Kandy district in the central highlands of Sri Lanka has an area of 1940.3 km^2^. Kandy city is in the center of the district. The estimated population of the district in 2015 was 1,416,000. Kandy is reported as a district with high leptospirosis incidence [7]. The Sri Lanka Department of Meteorology considers the data of the Katugastota weather station as representative of the Kandy district [15]. We used weather data from that weather station for our study. Figure 1 is a map showing the locations of our study area and that weather station.

We did the following estimates for our study period using the data of Katugastota weather station. The average annual rainfall was 2056 mm, average temperature was 25C° and average relative humidity was 83.7%.

### Objectives and hypotheses

Our general objective was to identify the correlation between the leptospirosis incidence and the weather of Kandy district and to explore the utility of the findings. Our specific objective was to determine the correlation (with the lag periods) between weekly leptospirosis incidence and the following weather parameters of Kandy district (Katugastota weather station) for the 2006–2015 period and then to explore the ways to use the information for improving leptospirosis control. Weekly rainfall, count of wet days per week (Sri Lanka Department of Meteorology defines a wet day as a day [24 hours] with rainfall > 1 mm), count of days with rainfall > 100 mm per week (Sri Lanka Department of Meteorology defines a day with rainfall 100–150mm as fairly heavy and > 150 mm as heavy rainfall [17]) and average temperature, minimum temperature and average relative humidity. Rainfall is known to influence transmission of leptospirosis and outbreaks were reported after heavy rains and flooding in the environment [1–6,11]. A larger number of wet days per week prevents the drying of soil and top-up and sustains small surface water collections, thus helping for the survival of leptospires in the environment. After heavy rains some leptospires in the soil get washed out to water ways utilized by people and to low-lying areas especially to rice paddies and rarely even to residences via flood water. Therefore, we expected positive correlations between leptospirosis incidence and peaks of rainfall, count of wet days per week and count of days with a rainfall > 100 mm per week. High humidity favors survival of leptospires in the environment and they get killed by desiccation when humidity is low [9]. Hence, we expected a positive correlation between relative humidity and leptospirosis incidence. Warm weather is reported to prolong the survival of leptospires in the environment [2–4]. Hot weather encourages certain activities like more bathing in the same shrinking body of water by people and animals [3]. Hence we expected a positive correlation between leptospirosis incidence and average temperature and minimum temperatures.

## Methods

### Data

Ours is a retrospective analytical study. We obtained the numbers (counts) of leptospirosis cases notified from the Kandy district each week by going through the weekly epidemiology reports of the Ministry of Health of Sri Lanka from 2006 to 2015. We bought the daily data of rainfall, minimum temperature, daytime and night-time humidity of the Katugastota weather station for the same period, from Sri Lanka Department of Meteorology. The annual estimated mid-year population of the Kandy district for 2006–2015 was obtained from the Sri Lanka Department of Census and Statistics.

### Analysis

We converted daily weather data to weekly data. We have estimated weekly leptospirosis incidence per 100,000 population using notified cases and mid-year population data. We drew time series graphs and looked for any visually observable correlation between the changes of averages of weekly weather parameters and averages of weekly leptospirosis incidences during the course of the 52 weeks of the year, for 2006–2015.We considered the averages of leptospirosis incidences and weather parameters of the first week of all 10 years as the average for the first week, and the same for the following 51 weeks of the year when making these graphs.

Wavelet time series analysis (wavelet analysis) was employed as our core method to establish the association between the weekly leptospirosis incidence and weekly weather parameters and to determine the lead/lag periods. In wavelet analysis, we select an appropriate window and it is shifted along the signal in a time series, and for every position we calculate the wavelet spectrum. This process is then repeated many times with a slightly shorter and longer window for every new cycle. With wavelet transform, the result will be a collection of time-frequency representations of the signal with different resolutions. Cross wavelet transform (XWT) and wavelet coherence (WTC) can be used for examining relationships in time-frequency space between two time series. While continuous wavelet transform (CWT) is a common tool for analyzing localized intermittent oscillations in a time series, it is very often desirable to study two time series together that are expected to be correlated. The XWT locates regions in time-frequency space with high common power. WTC is the square of the cross spectrum normalized by the individual power spectra. WTC gives a quantity between 0 and 1 and measures the cross correlation between two time series as a function of frequency. The details of the methodology are the same as the methodology of our study, ‘The Interrelationship Between Dengue Incidence and Diurnal Ranges of Temperature and Humidity in a Sri Lankan City and Its Potential Applications’, published in this journal [15]. Additional details of wavelet analysis of a time series and the definitions we have used can be found in the following references [18–20].

## Results

A total of 1573 leptospirosis cases were reported during our study period of 10 years from the Kandy district. The average annual leptospirosis incidence for the district was 11.2 per 100,000 population.

**Figure 2.**
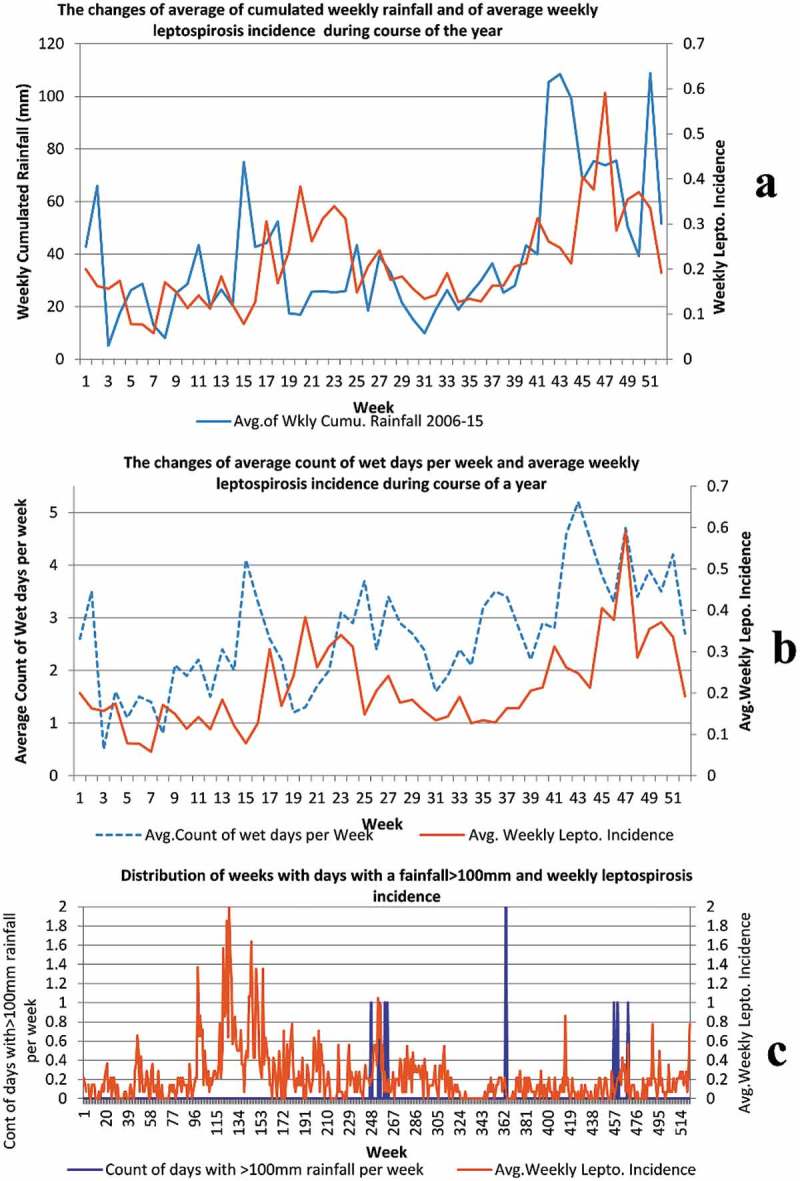
Changes of averages of weekly rain parameters and of average weekly leptospirosis incidence during the course of the year, for 2006–2015. Panel 2a: Changes of average of cumulated weekly rainfall (in millimeters) and of average weekly leptospirosis incidence (per 100,000 population) during the course of the year, for 2006–2015. x-axis: weeks/primary y- axis: average of cumulated weekly rainfall/secondary y-axis: average weekly leptospirosis incidence (per 100,000 population). Panel 2b: Changes of average count of wet days (days with rainfall > 1 mm) in a week and of average weekly leptospirosis incidence (per 100,000 population) during the course of the year, for 2006–2015. x-axis: weeks/primary y- axis: average of count of wet days per week/secondary y-axis: average weekly leptospirosis incidence (per 100,000 population).Panel 2c: shows the distribution 8 weeks with days with a rainfall > 100 mm and weekly leptospirosis incidences during the course of the 552 weeks of our study period. x-axis: week/primary y-axis: weeks with days with a rainfall > 100 mm/secondary y-axis: weekly leptospirosis incidence (per 100,000 population).

Figure 2 consists of time series graphs of three rain parameters and leptospirosis incidence.

Figure 2 consists of three panels. Panel 2a shows the changes of average cumulated weekly rainfall and average of weekly leptospirosis incidences during the course of the 52 weeks of the year, for 2006–2015. It depicts periods of low rainfall that were associated with low leptospirosis incidence and periods of high rainfall were associated with high leptospirosis incidence after a lag period. Panel 2b shows the changes of the average count of wet days (count of days with rainfall > 1 mm) in a week and the average of weekly leptospirosis incidences during the course of the 52 weeks of the year, for 2006–2015. From 42–52 weeks of the year the wet days per week remain high (generally > 3 days per week) and it is followed by a similar rise of leptospirosis incidence. Generally the temporal sequence of wet days per week is followed by leptospirosis incidence with a lag. Nonetheless following frequent wet days in weeks 23–28 there is no rise of leptospirosis incidence. When the count of wet days drops below 2 per week in weeks 3–9, a drop of leptospirosis incidence ensues after a lag. Panel 2c is different from other panels and shows the distribution of 8 weeks with days with a rainfall > 100 mm and weekly leptospirosis incidences during the course of the 552 weeks of our study period. Peaks of leptospirosis incidence are seen following weeks with days with rainfall > 100 mm. However there are frequent peaks and troughs of leptospirosis incidence and we cannot see a clear association between the two time series of panel 2c.

**Figure 3.**
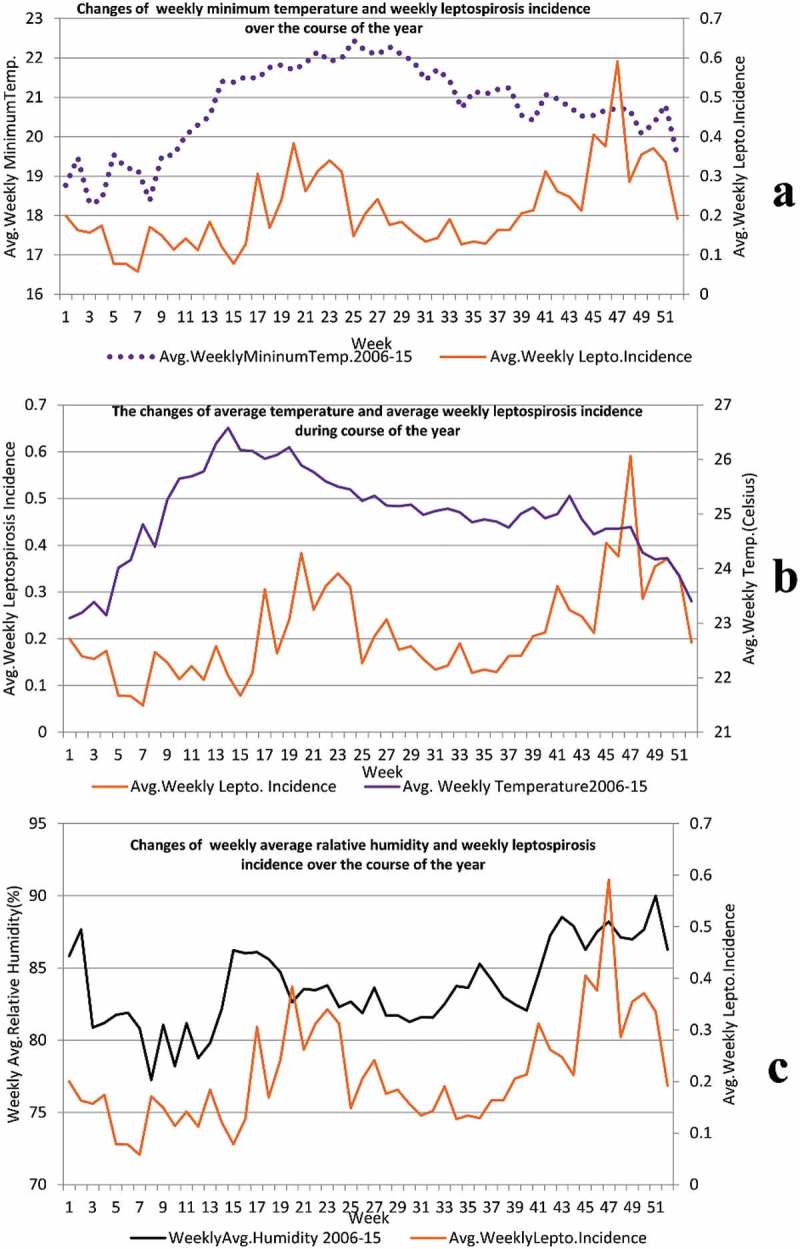
Panel 3a: Changes of average weekly temperature and of average weekly leptospirosis incidence during the course of the year, for 2006–2015. x-axis: weeks/primary y-axis: average weekly temperature (in C°)/secondary y-axis: average weekly leptospirosis incidence (per 100,000 population). Panel 3b: Changes of average of weekly minimum temperature and of average weekly leptospirosis incidence during the course of the year, for 2006–2015. x-axis: weeks/primary y- axis: average of weekly minimum temperature (in C°)/secondary y-axis: average weekly leptospirosis incidence (per 100,000 population). Panel 3c: Changes of average of weekly relative humidity and of average weekly leptospirosis incidence during the course of the year, for 2006–2015. x-axis: weeks/primary y- axis: average of weekly relative humidity (%)/secondary y-axis: average weekly leptospirosis incidence (per 100,000 population).

Figure 3 consists of time series graphs of the correlation of temperature and humidity with leptospirosis incidence.

Panel 3a illustrates the changes of average weekly temperature (in C°) and of average weekly leptospirosis incidence (per 100,000 population) during the course of the year, for 2006–2015. During the first 14 weeks of the year the average temperature rises gradually and it remains high and almost static in the 14th to the 20th week. Following a lag period, a corresponding rise of leptospirosis incidence is seen. From the 20th to the 25th week, the average temperature drops and then remains generally at the same level until the 42nd week. At the end of the year, average temperature drops even further but there is a peak of leptospirosis incidence. Panel 3b depicts the changes of average of weekly minimum temperature (in C°) and of average weekly leptospirosis incidence (per 100,000 population) during the course of the year, for 2006–2015. From the last week of the year until the 10th week of the next year, minimum temperature remains low (< 20°C) and there leptospirosis incidence is also low following a lag period. During the mid-year the minimum temperature remains > 21°C from week 15 to week 39. The leptospirosis incidence rises and then drops during this period. Minimum temperature gradually drops during the latter part of the year but leptospirosis incidence rises. In panel 3c we can see the changes in the average weekly relative humidity (%) and of average weekly leptospirosis incidence (per 100,000 population) during the course of the year, for our study period. Leptospirosis incidence follows the same pattern of temporal changes of average weekly humidity after a lag period.

**Figure 4.**
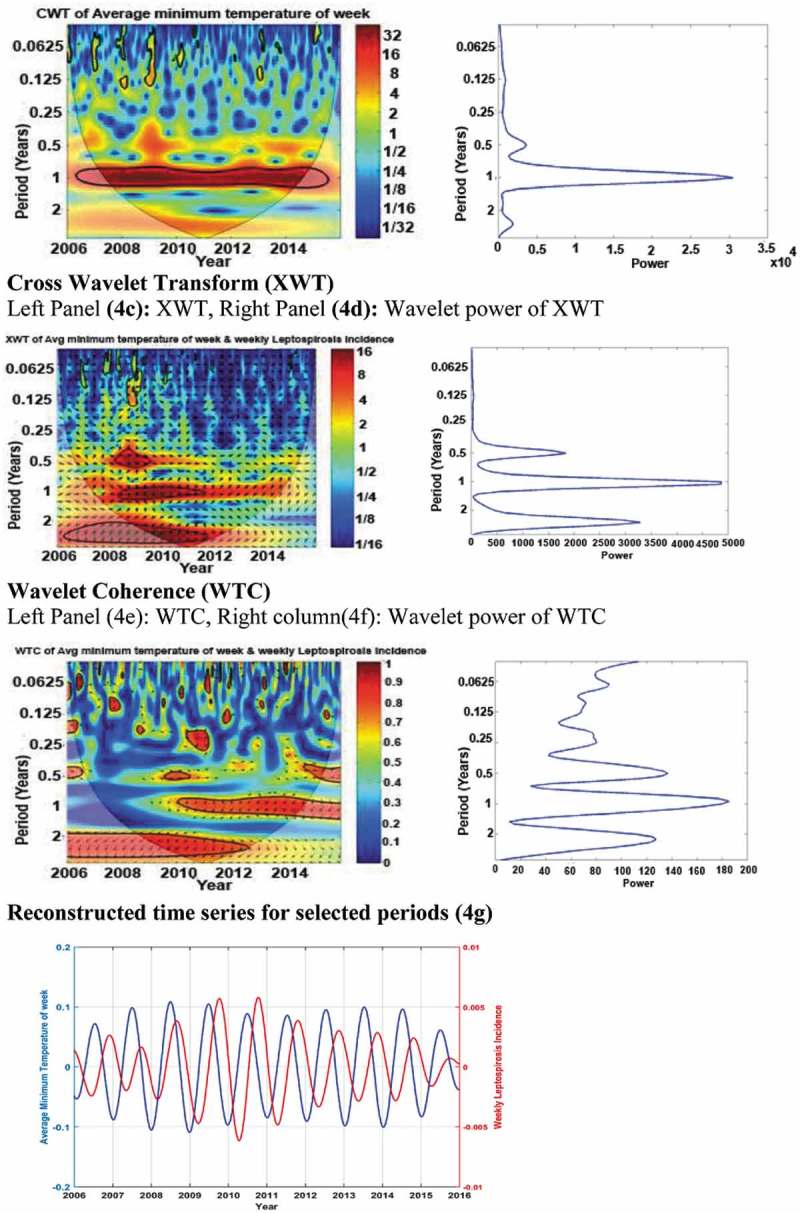
Results of wavelet analyses of weekly minimum temperature versus weekly leptospirosis incidence for 2006–2015: (Panel 4a) continuous wavelet transform (CWT) variations; (Panel 4b) wavelet power of CWT; (Panel 4c) crosswavelet transform (XWT) variations; (Panel 4d) wavelet power of XWT; (Panel 4e) wavelet coherence (WTC); (Panel 4f) wavelet power of WTC; and (Panel 4g) reconstructed time series for 2006–2015 period.

Figure 4 portrays the results of wavelet analysis of weekly minimum temperature versus weekly leptospirosis incidence for 2006–2015 as an example of our wavelet analysis results.

Panel 4a of Figure 4 illustrates the continuous wavelet transform of weekly minimum temperature, which expands the time series into time frequency space, while panel 4b summarizes the power for each period. Panel 4c shows the cross wavelet transform of weekly minimum temperature with weekly leptospirosis incidence, whereas panel 4d illustrates the power for each period. According to panels 4e and 4f, wavelet coherence is greatest between weekly leptospirosis incidence and weekly minimum temperature for yearly (period) cycles. The term ‘period’ in the vertical axis in panels 4a to 4f indicates the duration of a cycle (in years). There are color-coded panels on the right side of panels 4a, 4c and 4e. Those illustrate the magnitudes of CWT, XWT and WTC, in which dark blue and dark red indicate the lowest and highest, respectively. The thin U-shaped black lines in 4a, 4c and 4e are the cone of influence which demarcates the area not influenced by edge effects in wavelet analysis. The thick black lines in panels 4a, 4c and 4e are the 5% significance level using the red noise signal model. The arrows in panels 4c and 4e are vectors indicating the phase difference. A horizontal arrow pointing from left to right signifies in the phase and an arrow pointing vertically upward means the second series lags behind the first by 90°.

Panel 4g is the reconstructed time series for the period of study (2006–2015).The average time period between peaks of weekly minimum temperature and subsequent peaks in the weekly leptospirosis incidence in this time series was 13 weeks and that is the average lag period.

Table 1 gives the summary of all of our wavelet analysis results.

**Table 1.** Summary of the results of wavelet analysis.

Weekly weather parameter	The average lag periods in weeks and the range of lag periods is within brackets	Correlation with weekly leptospirosis incidence
Rainfall (in millimeters)	1 *(1–2)*	Nadirs (troughs) of rainfall were followed by a nadirs of leptospirosis incidence
Count of days with rainfall > 1 mm (wet days) in a week	2 *(0–6)*	Peaks of the count of wet days were followed by peaks of the leptospirosis incidence
Number of days with rainfall > 100 mm in a week	3 *(1–5)*	Only 8 such weeks for 10-yr study period (in 2010, 2011, 2012 and 2014). Peaks of such weeks were followed by peaks of leptospirosis incidence
Average temperature (in C°)	20 *(14–25)*	Peaks of average temperature were followed by peaks of leptospirosis incidence
Minimum temperature (in C°)	13 *(9–20)*	Peaks of minimum temperature were followed by peaks of the leptospirosis incidence
Average relative humidity (%)	1 *(0–2)*	Peaks of average relative humidity were followed by peaks of the leptospirosis incidence

## Discussion

Time series graphs of our Figure 2 show a general rise of leptospirosis incidence as we expected following high rainfall and frequent wet days and vice versa. Nevertheless, this correlation is not seen during some months of the year. Time series graphs of panels (3a) and (3b) of Figure 3 depict low leptospirosis incidence following weeks with low minimum and average temperatures as we anticipated. However, in contrast to our expectations despite a drop in average temperature during the last eight weeks of the year, leptospirosis incidence peaks after a lag. As panel 3c illustrates, leptospirosis incidence generally follows the temporal sequence of average humidity pattern in Kandy district. Leptospirosis incidence is the result of the net effect of all weather parameters and non-weather factors like risky behavior by people, and sanitation [5]. That explains the unexpected findings. Interpretation of weather and leptospirosis correlation patterns ignoring the effects of other factors gives only an incomplete picture of the real-world situation. This is true for the correlation between weather parameters and some other infections like dengue and even non-infective conditions like wheezing [15,21]. Below we explain an unexpected finding to illustrate that important point. During the last four months of the year, despite dropping average and minimum temperatures, leptospirosis incidence rises. This can be attributed to the simultaneous influence of other factors favorable for leptospirosis transmission. Weekly rainfall is high during this period with frequent wet days due to the second inter-monsoon and the North East monsoon rainy season; additionally, the average humidity also remains high. Preparation of fields and cultivation of rice and other crops of the ‘Maha’ season (main cultivation season) happen during this period. Working in rice paddies is considered as the main risk factor for contracting leptospirosis in Sri Lanka [22,23]. The disease is known as rice field fever in some countries [9]. Almost all local people work in their rice paddies in bare feet and in other fields wearing rubber slippers or barefoot, and most local home gardeners, rice and other crop cultivators, dairy farmers and construction workers work bare-handed and that is a common local risk factor of leptospirosis. The percentages of the Kandy district labor force engaged in agriculture in 2008 and 2012 respectively were 27.5% and 23% [24]. Moreover, many people that engage in other occupations also cultivate their home garden and rice paddy part-time. Bathing in waterways is also a risk factor for leptospirosis [6,23]. Many people, after working in rice paddies, wash themselves at a nearby waterway that irrigates those rice paddies. The addition of these factors explains the rise of leptospirosis during the last four months of a year. The mid-year rise of the leptospirosis incidence can be explained by favorable rain, temperature and humidity parameters for leptospirosis transmission plus the ‘Yala’ season of cultivation which coincides with the South West (Indian summer) monsoon.

We give greater emphasis to our wavelet analysis results due to reasons given in the background section. The results summarized in Table 1 are as we have expected and confirm the influence of local weather on leptospirosis incidence in Kandy.

The annual national leptospirosis incidence is 5.4 per 100,000 population and incidences range from approximately 0.1 to 10 per 100,000 per year globally [6,25]. The average annual leptospirosis incidence for Kandy district for our period of study was 11.2 per 100,000 population, which is above national and global averages. That is despite an active health ministry preventive program, especially after the 2008 outbreak. The 2008 outbreak was the worst recorded outbreak in Sri Lanka and that affected Kandy and several other districts in the wet zone of Sri Lanka [6]. In 2008 the incidence for Kandy district was 33.9 per 100,000 population according to our estimates and the national incidence was 37.5per 100,000 population [6].

We believe that the local weather is conducive to the survival of leptospires in the environment and has contributed to high incidence in Kandy district as we will explain below. The average annual rainfall for our period of study was 2056 mm, the average wet days per year was 140 and the average relative humidity was 83.7%. Such conditions help to maintain moisture in soil and surface water collections. There were only 9 days that had a rainfall > 100 mm during these 10 years. Nevertheless, there was no extreme rise of leptospirosis incidence following those. Such reported rises elsewhere have occurred mainly due to people getting contact with leptospires dispersed by flood water [3,5,11,12]. Kandy has many waterways compared to its area but due to its hill terrain storm water readily flows to waterways. Therefore floods affect small areas and are mild and short-lasting in Kandy compared to the flat lowlands of Sri Lanka. The Mahaweli, the longest river of Sri Lanka, flows through major population centers of Kandy district and a vast area of Kandy is a part of that river basin. Nevertheless, a few dams and reservoirs (constructed in the 1970s and 1980s) across the river prevent floods. However, minor and short-lived floods affecting fields, pathways and rarely residences occur due to over-flowing of streams after heavy rains. Paddy fields are usually situated in low-lying areas and usually get flooded after heavy rain.

Temperature is the limiting factor of the survival of leptospires in the environment in temperate climates and outbreaks usually occur in summer or fall [2]. Ambient temperatures < 7.1°C or > 34°C are detrimental to the survival of leptospires [13]. The lowest minimum temperature during our study period was 11.4°C and the maximum temperature rose to > 34°C only on 42 days during our study period of 10 years. The average temperature was 25°C. Those data indicate that the temperature of Kandy was also favorable for the survival of leptospires in the environment. Soil temperature (that influences the survival of leptospires in soil) and ambient temperature are not the same. However, a study conducted in an area with the same average temperature as in Kandy shows that they are strongly correlated even up to a depth of 10 cm [26].

The abundance and activity of pest rodent populations in tropics also depends on weather. Hot humid conditions, like in Kandy, favor the thriving of rodents [10,27]. Kandy is one of the districts in Sri Lanka with high livestock density [28]. Compared to rodents, these large mammals excrete large amount of leptospires when they have the disease [29]. A past study has demonstrated that a considerable percentage of dairy cattle and peri-domestic rodents around farms in Kandy are infected with a broad spectrum of leptospiral serogroups and half of these cattle graze in paddy fields [7].

Because of the hilly terrain and high population density of 730 people/km^2^ (in 2015) most of the farmlands in Kandy are small (46% of farms are < 0.1 hectares in area according to the Department of Census and Statistics) and most work is done manually. Cow dung has been a popular fertilizer in Sri Lanka for centuries and people apply it to crops manually. Cow dung usually gets contaminated with urine at cattle sheds. The abundance of small-scale farms in an area was identified as a risk factor for leptospirosis in Sri Lanka by a past study [23]. A small-scale study done in Kandy has demonstrated that 62% of house rats and roof rats trapped were sero-positive for leptospirosis [30]. We presume that those factors are also likely to have contributed to high leptospirosis incidence in Kandy despite the disease being under-diagnosed and under-reported (as we later elaborate) in Sri Lanka.

A study on monthly leptospirosis incidence in Sri Lanka for 2005–2009 showed a correlation with rainfall after a two-month lag in most areas in one past study and they did not study other meteorological parameters as we did [23]. A report from neighboring Kerala, India, shows a peak of leptospirosis cases after a 7–10 days lag, following a rainfall peak [31]. A Thai study showed that the monthly count of leptospirosis cases is correlated with the rainfall and temperature with long (8–10 months) lag periods [32]. A Malaysian study has recorded a positive correlation between the number of reported leptospirosis cases with the number of rainy days per month and monthly average temperature for the 2004–2012 period [33]. A study done in an urban setting in Manila, Philippines, for a 12-year period demonstrated that weekly rainfall was proportionately associated with the counts of hospital admission for leptospirosis after a 2- week lag [12]. A nine-year study conducted in the Republic of Korea demonstrated a rise of leptospirosis cases following a rise of minimum temperature, rainfall, relative humidity and increased solar radiation [10]. An 11-year study performed in Reunion Island showed a positive correlation between serologically confirmed leptospirosis cases counts and monthly rainfall, temperature and global solar radiation [34]. A five-year study done in Rio de Janeiro city, Brazil, demonstrated that leptospirosis is associated with heavy rainfall [35]. A study done in Jamaica 1992–2007 showed a peak of leptospirosis cases following peaks of rainfall [36]. The results of those past studies done in very different settings employing different methodologies generally agree with our findings. However, only very few of the past studies worldwide have considered multiple weather parameters like us.

Considering the favorable weather and other conducive conditions for leptospirosis transmission, and already high leptospirosis incidence, we believe more attention needs to be given to the primary prevention of leptospirosis in Kandy district, especially during periods with favorable weather for leptospirosis transmission; for example, during the last three months of the year. The further enhancement of existing health education programs to make the Kandy population, especially vulnerable communities, more aware of the disease and preventive measures is important for this.

### Limitations of the study

The reported leptospirosis cases we used for our study are only a fraction of all the cases because sub-clinical and most mild cases do not seek hospital care, hence are not investigated and thus not reported. And there are other local infective diseases like Hanta virus that can mimic leptospirosis and so a few of the reported cases may not be leptospirosis [10,37]. But these are rare. In Sri Lanka, leptospirosis is diagnosed if a patient has suggestive symptoms with a history of likely exposure to leptospires (such as recently working in a rice paddy) with suggestive physical examination findings (such as icterus, conjuntival suffusion) plus suggestive laboratory workup results such as thrombocytopenia with high leucocyte count, increased liver transaminases, bilirubin, serum creatinine and blood urea levels . The diagnosis of the great majority of the reported cases in Kandy and rest of Sri Lanka was not confirmed either by culture, PCR test or by serology, and thus can be categorized as probable cases of leptospirosis [38]. Nevertheless, the data we used is the best data available in the country for a retrospective study. There are local variations of weather within the Kandy district due to hilly topography. However, the main population mass lives closer to Katugastota weather station and it is the only nationally recognized principal weather station in the district. It is not possible to determine the magnitudes of correlations by wavelet analysis which is our mainstay analysis method.

## Conclusions

The leptospirosis incidence in Kandy district is high and multiple local weather variables are correlated with the leptospirosis incidence of Kandy. The local weather is conducive for leptospirosis transmission. Leptospirosis preventive work in Kandy district deserves more attention.

## Paper context

Local weather affects the survival of pathogenic leptospira bacteria in soil and surface water from which people become infected. We have studied the interrelationship between 6 local weather parameters and leptospirosis incidence of Kandy district, Sri Lanka, for 10 years. They were correlated with 1–20 weeks average lag periods. We found that leptospirosis incidence in the Kandy district is high and local weather is conducive to leptospirosis transmission.
